# Radiation dose reduction based on CNR index with low-tube voltage scan for pediatric CT scan: experimental study using anthropomorphic phantoms

**DOI:** 10.1186/s40064-016-3715-y

**Published:** 2016-12-01

**Authors:** Takanori Masuda, Yoshinori Funama, Masao Kiguchi, Naoyuki Imada, Takayuki Oku, Tomoyasu Sato, Kazuo Awai

**Affiliations:** 1 Department of Radiological Technology, Tsuchiya General Hospital, Nakajima-cho 3-30, Naka-ku, Hiroshima, 730-8655 Japan; 2 Department of Diagnostic Radiology, Tsuchiya General Hospital, Nakajima-cho 3-30, Naka-ku, Hiroshima, 730-8655 Japan; 3 Department of Medical Physics, Faculty of Life Sciences, Kumamoto University, Kumamoto, Japan; 4 Department of Diagnostic Radiology, Graduate School of Biomedical Sciences, Hiroshima University, Hiroshima, Japan

**Keywords:** Contrast-to-noise-ratio (CNR), Low-tube voltage, Radiation dose, Image noise

## Abstract

**Background:**

To figure out the relationship between image noise and contrast noise ratio (CNR) at different tube voltages, using anthropomorphic new-born and 1-year-old phantoms, and to discuss the feasibility of radiation dose reduction, based on the obtained CNR index from image noise. We performed helical scans of the anthropomorphic new-born and 1-year-old phantoms. The CT numbers of the simulated aorta and image noise of the simulated mediastinum were measured; then CNR was calculated on 80, 100, and 120-kVp images reconstructed with filtered back projection (FBP) and iterative reconstruction (IR). We also measured the center and surface dose in the case of CNR of 14 using radio-photoluminescence glass dosimeters.

**Results:**

The CT number of the simulated aorta was increased with decreasing tube voltage from 120 to 80 kVp (362.5–535.1 HU for the new-born, 358.9–532.6 HU for the 1-year-old). At CNR of 14, the center dose was 0.4, 0.6 and 0.9 mGy at FBP and 0.5, 0.6 and 0.9 mGy at IR and with the new-born phantom acquired at 80, 100 and 120 kVp, respectively. The center dose for FBP image was reduced by 56% at 80 kVp, 34% at 100 kVp for the new-born and 36% at 80 kVp, 22% at 100 kVp for the 1-year-old compared with that at 120 kVp. We obtained a relationship between image noise and CNR at different tube voltages using the anthropomorphic new-born and 1-year-old phantoms.

**Conclusion:**

The use of index of CNR with low-tube voltage may achieve further radiation dose reduction in pediatric CT examination.

## Background

Pediatric CT examination plays an important role in establishing the diagnosis, interventional management for pediatric patients. However, the most serious drawback of CT is radiation exposure which links to an increased risk of cancer (Preston et al. 2007, 2012; Miglioretti et al. 2013; Mathews et al. 2013). Children are especially more susceptible to radiation effects than adults; therefore, minimization of the radiation dose is the critical issue in pediatric CT examinations (Brenner et al. 2001).

To reduce radiation dose for pediatric patients, automatic tube current modulation (ATCM) or combination of ATCM and automatic tube voltage selection (ATVS) are an available option for modern CT scanners (Lee et al. 2012; Mayer et al. 2014). As iodinated contrast material is frequently used at pediatric cardiac CT examination, we think that the combined ATVS and ATCM technique is more useful than single use of ATCM because iodine enhancement is improved at low-tube voltage scan. In the combined ATVS and ATCM technique, contrast-to-noise ratio (CNR) is adequate as an image quality index instead of image noise [standard deviation (SD) of the CT number] because identification or characterization of lesions depends on balance between image noise and contrast.

Currently, ATVS has not become popular while most CT scanners are equipped with ATCM. ATCM controls tube current only according to preset image noise irrespective of CNR. Even if the CT scanner is not equipped with ATVS, if we can identify the relationship between image noise and CNR at different tube voltages, we can utilize CNR index by converting image noise value to CNR value.

The purpose of our study was to figure out the relationship between image noise and CNR at different tube voltages using the anthropomorphic new born and 1-year-old phantoms at pediatric CT examinations and then to discuss the feasibility of radiation reduction based on the CNR index and low-tube voltage.

## Methods

### Phantoms

**Fig. 1:**
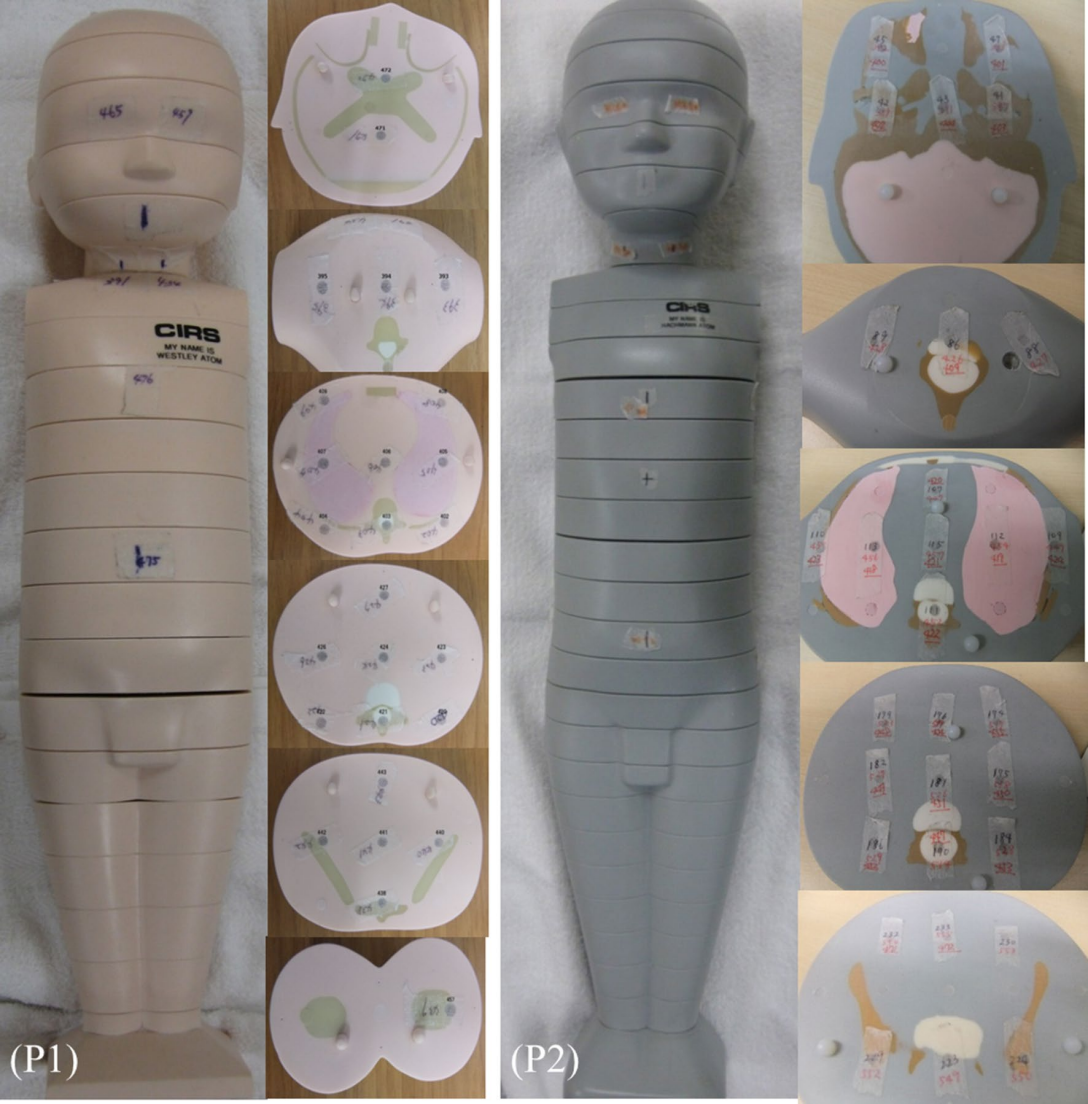
Anthropomorphic new-born (*left*) and 1-year-old (*right*) phantoms

We used two pediatric anthropomorphic phantoms (ATOM Phantom, CIRS, Norfolk, Virginia, USA) that represent the average individual as new born and 1-year-old child in the study (Fig. 1). Assumed body weight (BW) and body height (BH) for the new-born and 1-year-old phantoms are 3.5 kg and 51 cm and 10 kg and 75 cm, respectively. The phantoms were made of radiologically sensitive tissue-equivalent material and internally artificial skeletons, lungs, and soft tissue formulated for accurate simulation of medical exposures.

**Fig. 2:**
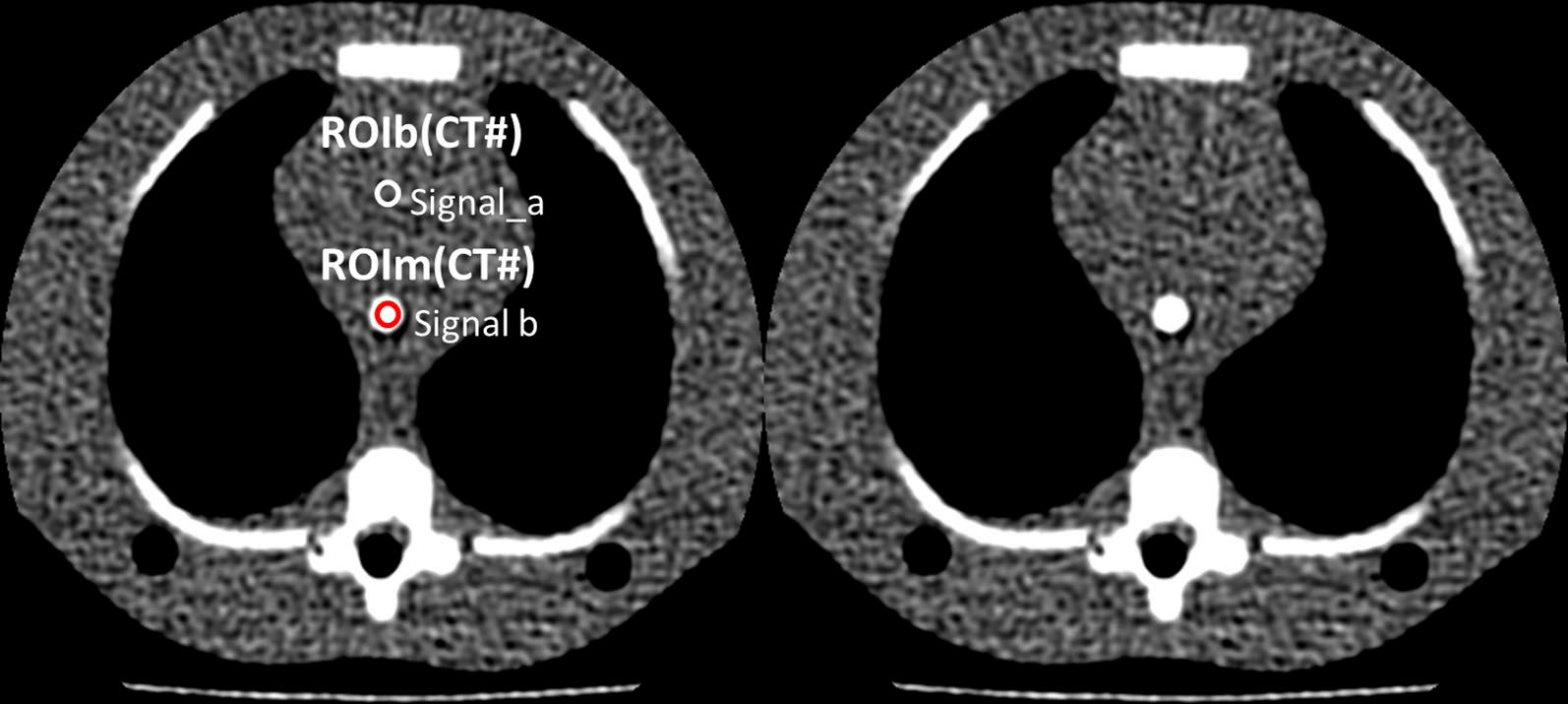
CT number of the simulated aortic (Signal_a) and the mediastinum portions (Signal_b) were measured within 10-pixel-diameter circular region of interest (ROI) in the each phantom

The phantoms contained a central tunnel of 5.0 mm in diameter for simulation of the blood-filled aorta (Fig. 2). We filled the simulated aortic lumen with diluted iodinated contrast material (CM) (Omnipaque-300; Daiichi-Sankyo, Tokyo, Japan) and adjusted the CT number with the 1-year-old phantom to 350–370 HU at 120 kVp based on our clinical experience; applied the same diluted iodinated CM to the new-born phantom (Fig. 2).

### CT scanning

All CT scans were performed on a 64-detector row CT scanner (Lightspeed VCT; GE Healthcare, Milwaukee, Wisconsin) including entire chest of the pediatric anthropomorphic phantom. The scanning parameters were as follows: scan mode, helical scan; section thickness, 0.625 mm; pitch factor, 1.375; gantry rotation time, 0.4 s; detector collimation, 64 × 0.625 mm; scan field of view, 100 mm for new-born phantom and 150 mm for 1-year-old phantom; matrix size, 512 × 512; and reconstruction kernel (standard; GE Healthcare). It is generally acknowledged that faster scanning speed and high pitch factor are necessary to obtain the pediatric CT images without motion artifact (Lell et al. 2011; Long et al. 2015).

The applied tube-voltages were 80, 100, and 120 kVp and tube current was set from 10 to 150 mA on a 10 mA step in two phantoms, respectively. CT images were acquired with filtered back projection (FBP) and iterative reconstruction (IR, blending of 30% of ASIR with FBP) algorithms under the standard kernel/filter.

### CT number and image noise measurement

We measured the CT number (HU) and image noise in the aortic (CT#_a_) and mediastinum portions (CT#_m_) of the phantoms using a circular region of interest (ROI; Fig. 2). The diameters of the circular ROIs acquired at the simulated aortic and mediastinum portions were all 4.0 mm. We acquired 100 consecutive images in the z direction at each scan, performed 3 scans, and then calculated mean values for all the phantoms. In our study, the contrast for calculation of CNR was defined as the value of aortic CT number in the images of 80, 100, and 120-kVp in each phantom and CNR was calculated from CT number of the simulated aorta divided by image noise (aortic CT number/image noise) at the simulated mediastinum portion. Finally we obtained the relationship between image noise, CNR and tube current and then also obtained the relationship between image noise and CNR. We calculated the image noise during optimal CNR using lower tube voltage at clinical practice.

### Dose measurements

We performed helical scans for calculating tube current at 80, 100 and 120 kVp during CNR of 14 using new-born and 1 year-old phantoms. We measured the surface and center doses in the new-born and 1-year-old phantoms. The radio-photoluminescence glass dosimeters (RPLGD) GD-352M with a tin filter (Dose Ace, glass dosimeter; Asahi Techno Glass, Shizuoka, Japan) were inserted at the positions of the phantom center and front, back, left, and right side of the phantom surface. We performed 3 scans from the upper end of the apex to the lower region of the diaphragm in the pediatric cardiac CT scan. The FDG-1000 reader (ATGC-2004; Asahi Techno Glass) were used for dose measurements.

## Results

### Aortic CT number

The aortic CT numbers with FBP and IR increased for new born from 363 to 535 HU with FBP and from 362 to 534 HU with IR from 120 to 80 kVp. And for 1 year old they increased from 359 to 532 HU with FBP and from 361 to 533 HU with IR for 120–80 kVp on scans of pediatric anthropomorphic phantoms (Table 1). The iodinated contrast improvement acquired at 80 and 100 kVp was approximately 1.5 and 1.2 times higher than the value of 120 kVp irrespective of the pediatric phantom size and reconstruction algorithm of FBP or IR.

**Table 1:** Aortic CT numbers for anthropomorphic pediatric phantoms acquired at 80, 100, and 120 kVp

Tube voltage (kVp)	CT number of aorta (HU)		CT number of mediastinum (HU)	
	New born	1 year old	New born	1 year old
FBP				
80	535 (519–541)	531 (525–535)	13 (11–14)	22 (20–25)
100	443 (435–451)	438 (425–440)	14 (11–15)	27 (24–31)
120	363 (358–367)	358 (357–360)	16 (12–17)	30 (28–33)
IR				
80	533 (522–543)	532 (528–539)	12 (10–13)	21 (19–23)
100	433 (428–438)	430 (423–436)	13 (11–14)	26 (24–30)
120	362 (359–365)	362 (359–363)	15 (13–16)	29 (27–32)

### Variation of image noise and CNR

**Fig. 3:**
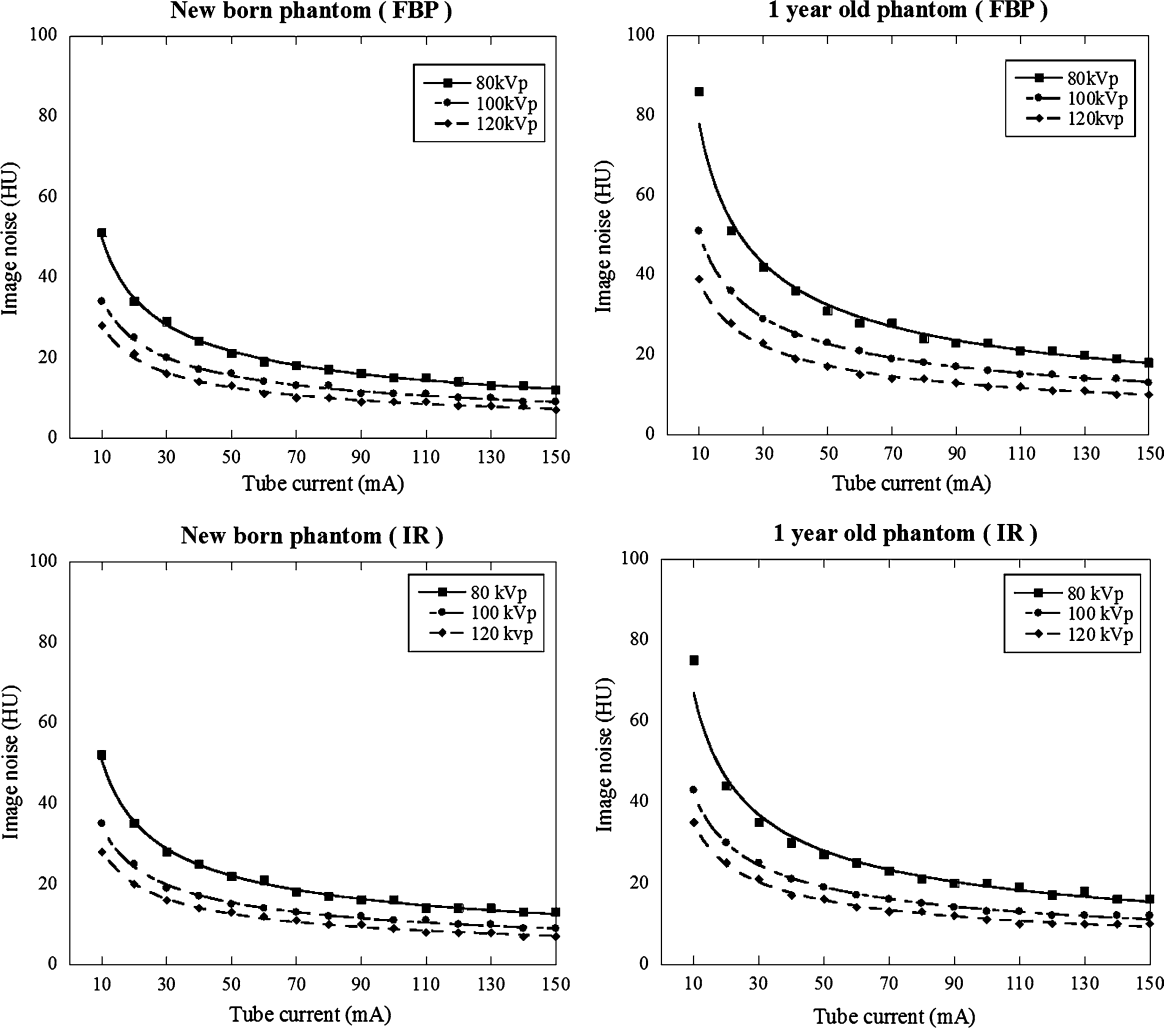
Image nose (SD of CT number) variations with varying tube current: new-born and 1-year-old phantoms

**Fig. 4:**
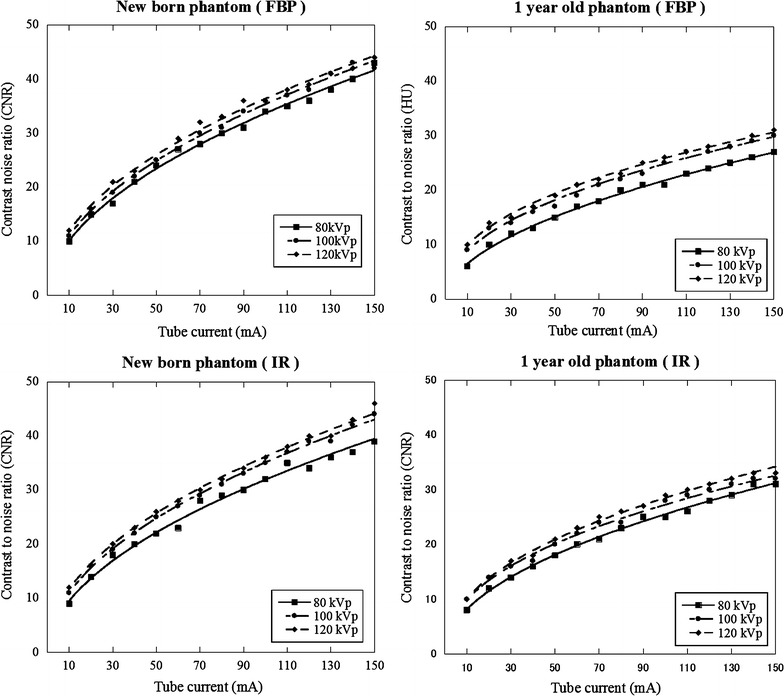
Variations of contrast-to-noise ratio (CNR) with varying tube current: new-born and 1 year-old phantoms

**Fig. 5:**
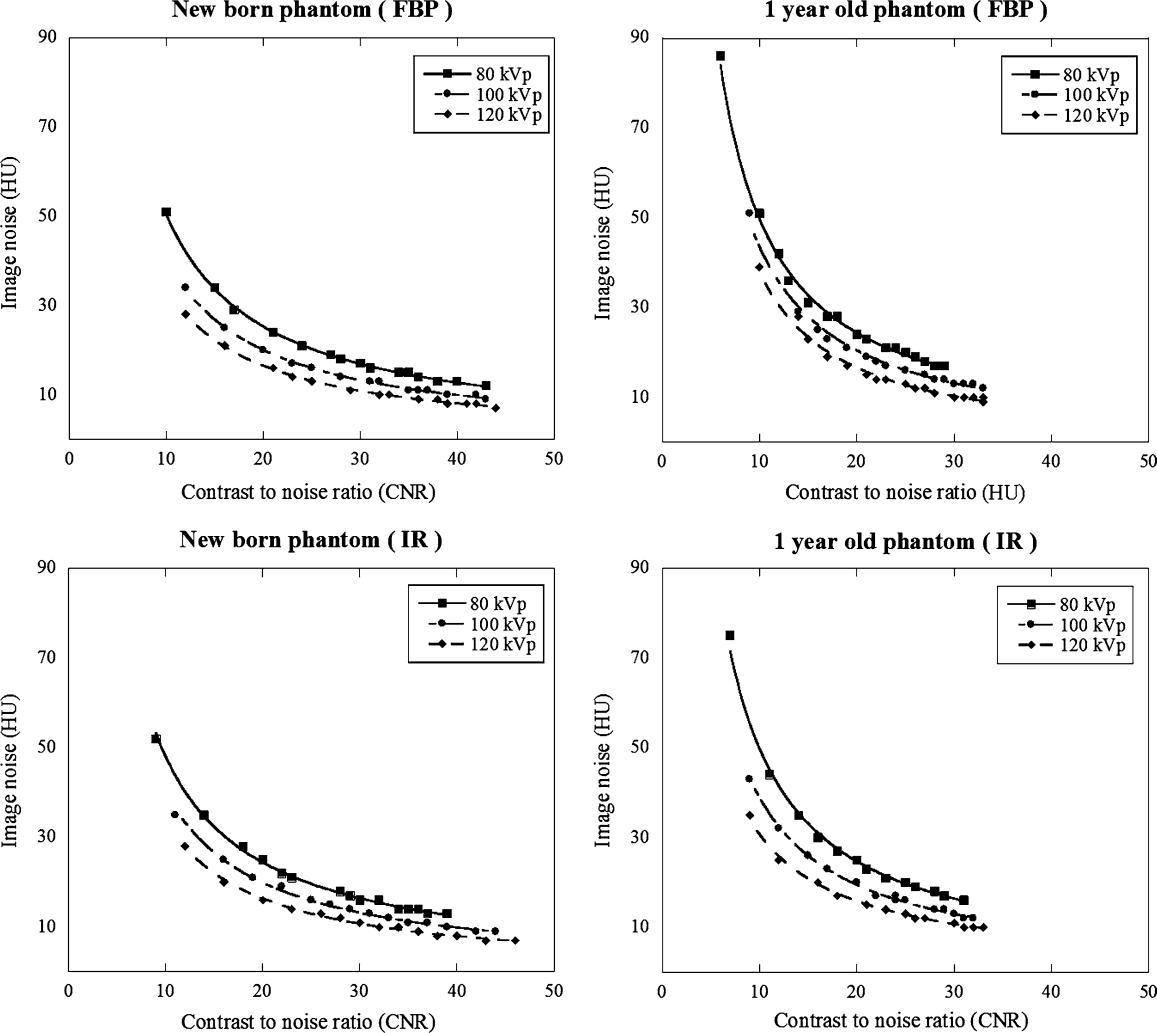
Relationship between image noise and contrast-to-noise ratio (CNR) at different tube voltage: new-born and 1-year-old phantoms

The variation for image noise or CNR with increasing tube current on new-born and 1-year-old phantoms was shown in Figs. 3 and 4. The variation curve for image noise entirely shifted higher image noise at 80 kVp compared with other tube-voltages at 100 and 120 kVp (Fig. 3). The variation curve for CNR was calculated from aortic CT number (Table 1) and image noise at different tube currents (Fig. 3). The relationship between CNR and image noise at different tube voltages was shown in Fig. 5. At CNR of 13, 14, and 15 with FBP and IR, image noise value acquired at 80 and 100 kVp was shown in Table 2 (also see Fig. 5). With increasing CNR from 13 to 15, image noise decreased approximately 6 HU at 80 kVp and 4 HU at 100 kVp.

**Table 2:** The image noise with low tube voltage for CNR at 13, 14, and 15

Tube voltage (kVp)	CNR		
	13	14	15
New born			
FBP			
80	39	37	33
100	32	30	26
IR			
80	39	37	34
100	31	30	27
1 year old			
FBP			
80	37	35	31
100	30	29	26
IR			
80	39	37	33
100	32	30	28

### Radiation dose

At CNR of 14, the tube current, image noise, surface, and center doses are listed in Table 3 (also see Figs. 3, 4, 5). The center dose was 0.4, 0.6 and 0.9 mGy with FBP and 0.5, 0.6 and 0.9 mGy with IR for new-born phantom and 0.9, 1.1 and 1.4 mGy with FBP and 0.7, 1.0 and 1.2 mGy with IR for 1-year-old phantom at 80, 100, and 120 kVp respectively. The center dose for FBP image was reduced by 56% at 80 kVp, 34% at 100 kVp for new born and 36% at 80 kVp, 22% at 100 kVp for 1 year old compared with the that at 120 kVp. The surface dose for FBP image was reduced by 33% at 80 kVp, 33% at 100 kVp for new born and 27% at 80 kVp, 13% at 100 kVp for 1 year old compared with the that at 120 kVp.

**Table 3:** Image noise and radiation dose at different tube voltages in the case of CNR at 14

Tube voltage (kVp)	Tube current (mA)	Surface dose (mGy)	Center dose (mGy)	Measurd image noise (HU)
New born				
FBP				
80	20	0.6	0.4	37
100	15	0.6	0.6	30
120	15	0.9	0.9	25
IR				
80	20	0.6	0.5	37
100	15	0.7	0.6	30
120	15	0.9	0.9	25
1 year old				
FBP				
80	45	1.1	0.9	35
100	30	1.3	1.1	29
120	25	1.5	1.5	25
IR				
80	40	0.9	0.7	37
100	25	1.1	1.0	30
120	20	1.4	1.2	25

**Fig. 6:**
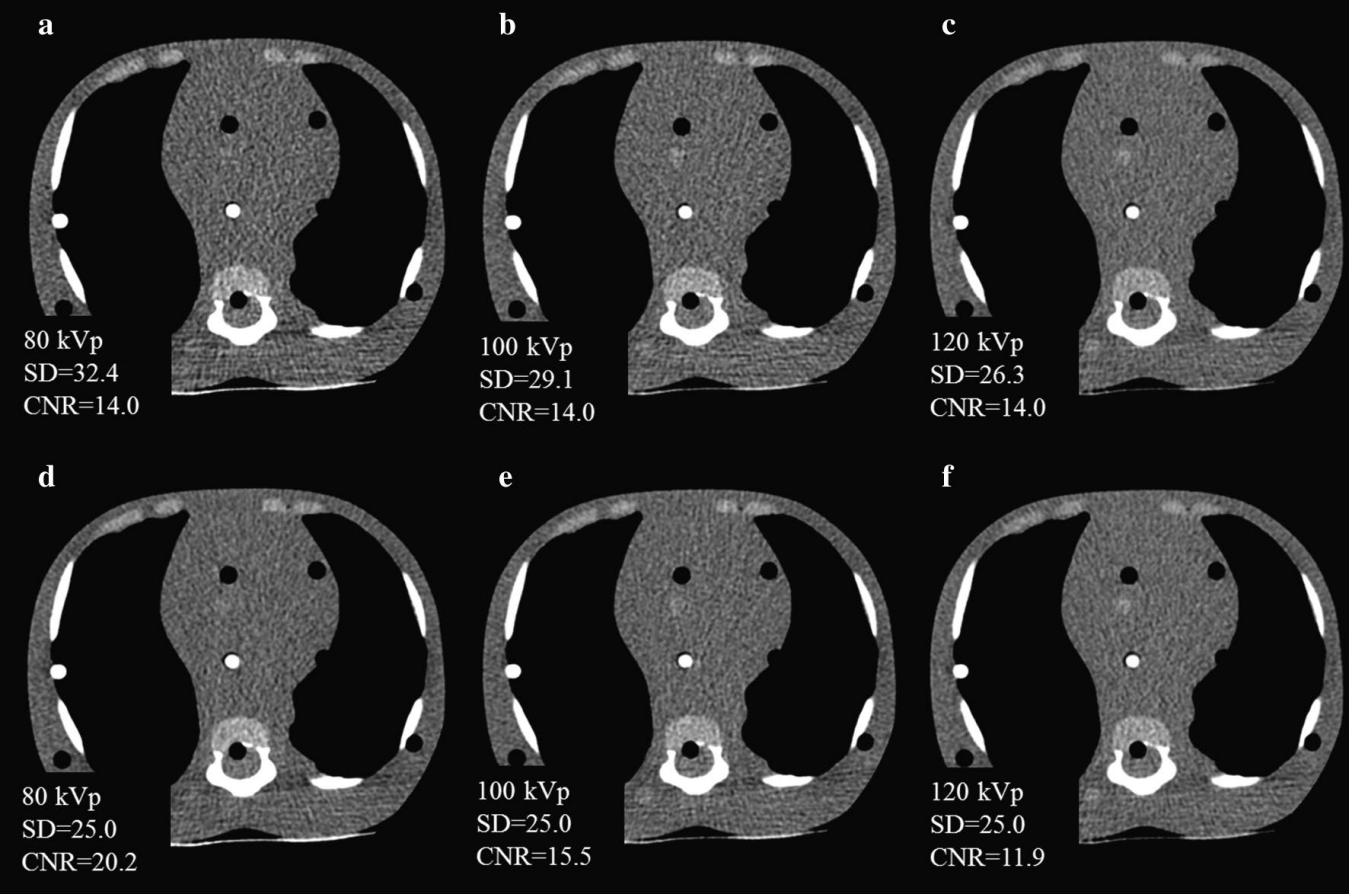
Anthropomorphic pediatric phantom image acquired for 1 year old at 80 (**a**), 100 (**b**) and 120 kVp (**c**) with CNR of 14, and at 80 (**d**), 100 (**e**) and 120 kVp (**f**) with image noise of 25 HU at IR

Figure 6 shows 1-year-old phantom images with FBP and IR at CNR of 14. The FBP and IR images acquired at 80- and 100 kVp were the same detectability as that at 120 kVp.

## Discussion

We obtained a relationship between image noise and CNR and evaluated the radiation dose using the index of CNR at different tube voltages on anthropomorphic new born and 1-year-old phantoms. At low-tube-voltage, the center dose based on the index of CNR was lower than that with the index of image noise at new born (e.g. 0.4 vs. 0.9 mGy with FBP and, 0.5 vs. 0.9 mGy with IR at 80 kVp) and 1-year-old phantoms (e.g. 0.9 vs. 1.5 mGy with FBP and 0.7 vs. 1.2 mGy with IR at 80 kVp) due to effect of increasing iodine contrast. The center dose for FBP image was reduced by 56% at 80 kVp, 34% at 100 kVp for new born and 36% at 80 kVp, 22% at 100 kVp for 1 year old compared with the that at 120 kVp.

When the index of CNR is applied, appropriate CT number is necessary to improve the vessel contrast (Boone et al. 2003; Siegel et al. 2004; Funama et al. 2005). Fei et al. (2008) reported that the best aortic CT number for cardiac examinations was over 350 HU at 120 kVp for maintaining image quality. Considering the reported data and our clinical experience, our study set the iodine contrast so that to 350 HU at 120 kVp. In the case of low-tube-voltage, the value increases 1.5 times at 80 kVp and 1.2 times at 100 kVp, respectively than the value of 120 kVp. To predict aortic CT number, past CT images with follow-up examination may be utilized if the same injection method is employed. In addition, the use of test bolus scan with diluted contrast medium may be useful (Masuda et al. 2014). Because, a strong correlation was found between aortic CT numbers on images obtained with a test bolus using diluted contrast material and subsequently acquired coronary CTA images.

When we use automatic tube current modulation for calculation of CNR, it is important to set the optimal noise index to avoid image quality degradation using ATCM. Considering the reported data and our clinical experience, image noise is approximately 25–30 HU achieved at 120 kVp (Layritz et al. 2013). Therefore, we set the image noise of 25 HU at 120 kVp and corresponding CNR was 14 (350/25). In clinical situation, we think the value of CNR ranged 12–16 is considered to be an adequate CNR. When low-tube-voltage is applied, the setting of the noise index must take into account the image contrast and noise. From our study, if we obtain the image of CNR at 14 and 80 kVp, setting image noise is 37 HU with FBP and IR for new born, 35 HU with FBP and 37 HU with IR for 1-year old (see Table 2). In addition, in the case of different contrast value, image noise needs to multiply the factor of increasing or decreasing contrast ratio from the Fig. 5. For instance, expected contrast and CNR are 400 HU and 14 with FBP for 1 year old, image noise calculates as 35 × 400/350 = 40 HU. Visibility of structure in the images for new-born and 1-year-old phantoms was comparable to the image acquired with 120 kVp. We think this is due to small body size and not pronounced the image noise texture. In addition, despite of real human, simple configuration of the phantom may help the visibility of structure. We need to determine upper limit of the image noise without sacrificing image quality in clinical situation even though CNR is maintained.

Many researchers have studied the low-tube-voltage technique (Dong et al. 2012; Yu et al. 2011; Kim and Newman 2010; Reid et al. 2010). some of them reported ATVS technique which automatically controls both tube voltage and tube current (Ghoshhajra et al. 2013; Funama et al. 2013; Durand et al. 2014). Funama et al. (2013) reported that ROC analysis-based CNR for lesion detection, CNR-based AEC potentially provide image quality advantages for clinical implementation. Also, Krazinski et al. (2014) reported that automated tube voltage selection can operator-independently optimize cardiovascular CTA image acquisition parameters with improved image quality at reduced dose. When maintaining CNR with low-tube-voltage technique, higher iodine contrast leads to increasing image noise and regulates increase of excessive tube current. In contrast, low-tube-voltage technique increases tube current for keeping image noise level without consideration of higher iodine contrast compared with the tube current at 120 kVp. To control the increasing tube current, IR is promising method. Iterative reconstruction is advanced developments that show the potential to improve image quality, primarily by reducing image noise, despite low-dose CT protocols (Moscariello et al. 2011; Winklehner et al. 2011; Marin et al. 2010). In addition, ATCM is also the necessary tool to control an optimal tube voltage selection.

Our study has several limitations. First, we evaluated image quality only for pediatric patients using an anthropomorphic phantom but not on real pediatric patients. The influence of cardiac and respiratory motion or stair-step artifacts on the image quality was not taken into consideration. Second, we evaluated new-born and 1-years old phantoms for focusing of CHD in pediatric patients. With increasing age, the results might be varied due to increasing body size. Third, we only used a small scan field of view and corresponding small bowtie filter. Toth (2002) showed that variations in dose and image quality changes are higher with a small bowtie filter than a medium or large bowtie filter and we performed the study using a single model of CT scanner from a single vendor. The relation between tube voltage, image noise, radiation dose, reconstruction kernel, slice thickness, tube filtration, detector system, and phantom size may depend to some degree on CT scan specifications and vary among scanners.

## Conclusion

In conclusion, we obtained relationship between image noise and CNR at different tube voltages using the anthropomorphic new born and 1-year-old phantoms at pediatric CT examinations. The use of CNR index with low-tube voltage may achieve further radiation dose reduction in pediatric CT.
